# RNA-Seq profiling in peripheral blood mononuclear cells of amyotrophic lateral sclerosis patients and controls

**DOI:** 10.1038/sdata.2019.6

**Published:** 2019-02-05

**Authors:** Susanna Zucca, Stella Gagliardi, Cecilia Pandini, Luca Diamanti, Matteo Bordoni, Daisy Sproviero, Maddalena Arigoni, Martina Olivero, Orietta Pansarasa, Mauro Ceroni, Raffaele Calogero, Cristina Cereda

**Affiliations:** 1 Genomic and post-Genomic Center, IRCCS Mondino Foundation, Pavia, Italy; 2 Department of Biology and Biotechnology “L. Spallanzani”, University of Pavia, Pavia, Italy; 3 Department of Brain and Behavioral Sciences, University of Pavia, Pavia, Italy; 4 General Neurology, IRCCS Mondino Foundation, Pavia, Italy; 5 Department of Molecular Biotechnology and Health Sciences, Bioinformatics and Genomics Unit, University of Turin, Torino, Italy

**Keywords:** Molecular neuroscience, Long non-coding RNAs, Transcriptomics

## Abstract

Coding and long non-coding RNA (lncRNA) metabolism is now revealing its crucial role in Amyotrophic Lateral Sclerosis (ALS) pathogenesis. In this work, we present a dataset obtained via Illumina RNA-seq analysis on Peripheral Blood Mononuclear Cells (PBMCs) from sporadic and mutated ALS patients (mutations in *FUS*, *TARDBP*, *SOD1* and *VCP* genes) and healthy controls. This dataset allows the whole-transcriptome characterization of PBMCs content, both in terms of coding and non-coding RNAs, in order to compare the disease state to the healthy controls, both for sporadic patients and for mutated patients. Our dataset is a starting point for the omni-comprehensive analysis of coding and lncRNAs, from an easy to withdraw, manage and store tissue that shows to be a suitable model for RNA profiling in ALS.

## Background & Summary

Deep sequencing technologies allow high-throughput massive-parallel RNA sequencing, permitting an extensive characterization of transcriptomic profile of cell populations and tissues. RNA-seq analyses are the gold standard for in depth characterization of global changes in gene expression levels between different conditions (e.g.: disease and healthy subjects or treated and untreated samples)^1^.

Such comprehensive analysis are currently bringing the light on the expression of largely unexplored RNA classes. While coding RNA involvement in a wide plethora of disorders has been subject of intense research, the more recently revealed class of lncRNAs is only starting to be characterized. LncRNAs are RNA transcripts greater than 200 nucleotides that lack an open reading frame and therefore do not encode proteins^2^. The heterogeneity of lncRNAs, both in biological type and function, is a major obstacle that makes it difficult to extract information about lncRNAs function and to enrich our knowledge^3^. While coding genes are widely annotated, high-quality catalogues of lncRNAs and information on the tissues in which they are expressed are recently being constructed^4–6^. Recent efforts are directed to characterize this largely unexplored functional component of the genome. Deep sequencing is a precious instrument to obtain information suitable to investigate both coding and lncRNAs simultaneously in the same sample, and this comprehensive RNA analysis can elucidate fundamental mechanisms in gene expression regulation at post-transcriptional level^7,8^.

There is mounting evidence that altered RNA metabolism, both involving coding and non-coding RNAs, plays an important role in ALS pathogenesis^9–13^. ALS is an adult-onset, progressive and fatal neurodegenerative disease, caused by the selective loss of both upper and lower motor neurons in the cerebral cortex, brainstem and spinal cord. The pathogenesis of the disease is still unknown. Moreover, the investigation of ALS mechanism is challenging due to the difficulty to obtain a sufficient amount of biological material from ALS patients: it is indeed impossible to access biological samples from the most affected areas, such as spinal cord. PBMCs have been shown to be a convincing and realistic model for ALS, since many pathways, typically located in neurons, are also activated in these cells^8,14,15^.

Furthermore, low ALS incidence (ranging between 1 and 2.5/100’000)^16^ and low percentage of familial ALS cases (5-10%) impose the requirement of data sharing and data aggregation among different hospitals and laboratories in order to obtain a sufficient sample size. Detailed curation of these datasets is paramount for accurate interpretation, widespread dissemination, and repurposing of data.

In this paper, we present a dataset suitable for the analysis for both coding RNAs and lncRNAs in PBMCs of 15 sporadic ALS (sALS) patients, 9 ALS patients with mutation in classical ALS-genes (*SOD1*, *FUS*, *TARDBP* and *VCP*) and 7 healthy controls. More in detail, these data are suitable for antisense lncRNAs detection and characterization because of the chemistry of the protocol properly design to maintain strandness information. Strand-specific RNA-Seq permits a more complete understanding of the transcriptome, enhancing resolution for sense/antisense profiling^17^.

Part of this dataset was used to perform an analysis of differentially expressed transcripts (coding RNAs and lncRNAs)^8^ aiming at investigating the importance of sense and antisense RNA regulation in central and peripheral systems in ALS disease.

To our knowledge, no dataset of RNA expression in PBMCs from ALS patients is publicly available. Our dataset is a starting point for the omni-comprehensive analysis of RNA classes, from an easy to withdraw, manage and store tissue that shows to be a suitable model for RNA profiling in ALS^8^.

It was shown that in RNA-seq studies, increasing sample size is the best choice to enhance statistical power^18^. For these reasons, the re-usability of this dataset is related to the possibility to expand studies with smaller sample sizes, especially for mutated patients, to identify new sense and antisense transcripts with altered expression in both sporadic and mutated ALS patients compared to healthy controls and to provide an open-angle point of view for a broad-spectrum characterization.

## Methods

### Isolation of human PBMCs from ALS patients and healthy controls

#### Subject enlistment

24 ALS patients and 7 age- and sex-matched healthy controls (CTRL) were recruited after obtaining written informed consent (Table 1). ALS patients underwent clinical and neurologic examination at IRCCS National Neurological Institute “C. Mondino” (Pavia, Italy). All patients were diagnosed with ALS as defined by El Escorial criteria. All ALS patients were analysed for causative mutations in SOD1, TARDBP, FUS, C9orf72, ANG and VCP genes: 15 patients resulted to be sporadic (sALS) and are indicated as ALS-s1, ALS-s2 and so on. Three patients were mutated in SOD1 gene (SOD1-m1, SOD1-m2 and SOD1-m3), three in FUS gene (FUS-m1, FUS-m2 and FUS-m3), two in TARDBP gene (TARDBP-m1 and TARDBP-m2) and one in VCP gene (VCP-m1). The seven control subjects (indicatetd as CTRL1-CTRL7) have been recruited at the Transfusional Service and Centre of Transplantation Immunology, Foundation San Matteo, IRCCS (Pavia, Italy). All details are reported in Table 1.

The study protocol to obtain PBMCs from patients and controls was approved by the Ethical Committee of the National Neurological Institute “C. Mondino”, IRCCS (Pavia, Italy). Before being enrolled, the subjects participating in the study signed an informed consent form (Protocol n°375/04 – version 07/01/2004). All experiments were performed in accordance with relevant guidelines and regulations. These methods and following methods are expanded versions of descriptions in our related work^8^.

#### Isolation of human PBMCs

PBMCs were prepared by centrifugation. Peripheral blood was layered (density = 1.077) and centrifuged at 950 g for 30 min. After isolation on a Ficoll-Histopaque layer (Sigma Aldrich, Italy), cell viability was assayed by a trypan blue exclusion test and cells were then used for RNA extraction.

#### RNA extraction

Samples were homogenized and total RNA was isolated by Trizol® reagent (Life Science Technologies, Italy) following the manufacturer’s protocol. RNAs were quantified using a Nanodrop ND-100 Spectrophotometer (Nanodrop Technologies, Wilmington, USA) and a 2100 Bioanalyzer (Agilent RNA 6000 Nano Kit, Waldbronn, Germany); RNAs with a 260:280 ratio of ≥1.5 and an RNA integrity number of ≥8 were deep sequenced (Supplementary Figure 1).

#### RNA-seq library preparation, sequencing and analysis

Sequencing libraries were prepared with the Illumina TruSeq Stranded RNA Library Prep, version 2, Protocol D, using 500-ng total RNA (Illumina,USA). The qualities of the libraries were assessed by 2100 Bioanalyzer with a DNA1000 assay. Libraries were quantified by qPCR using the KAPA Library Quantification kit for Illumina sequencing platforms (KAPA Biosystems); RNA processing has been carried out using Illumina NextSeq 500 Sequencing, using 8 samples for each run mixing samples and controls in each flowcell to avoid not manageable batch-effects. FastQ files were generated via llumina bcl2fastq2 (Version 2.17.1.14 - http://support.illumina.com/downloads/bcl2fastq-conversion-software-v217.html) starting from .bcl files produced by Illumina NextSeq sequencer (see Table 2).

### Quality validation and read mapping

**Figure 1:**
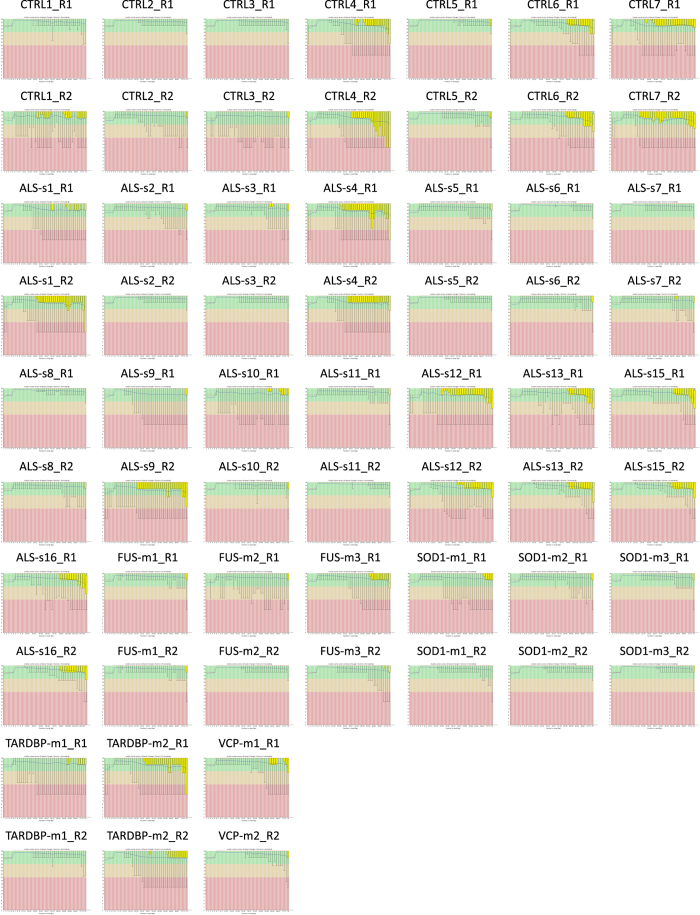
Quality assessment FASTQ data. Quality assessment of raw FASTQ sequence data for paired end and left (sample_name_R1) and right reads (sample_name_R2). Box and whisker plots demonstrate the distribution of per base quality for each left and right read position read for each of the analyzed samples. Mean value is indicated by the blue line and the yellow box represents the interquartile range (25–75%) with the lower and upper whiskers represent the 10 and 90% points, respectively. Plots were generated by FastQC program (see Code Availability 1).

**Figure 2:**
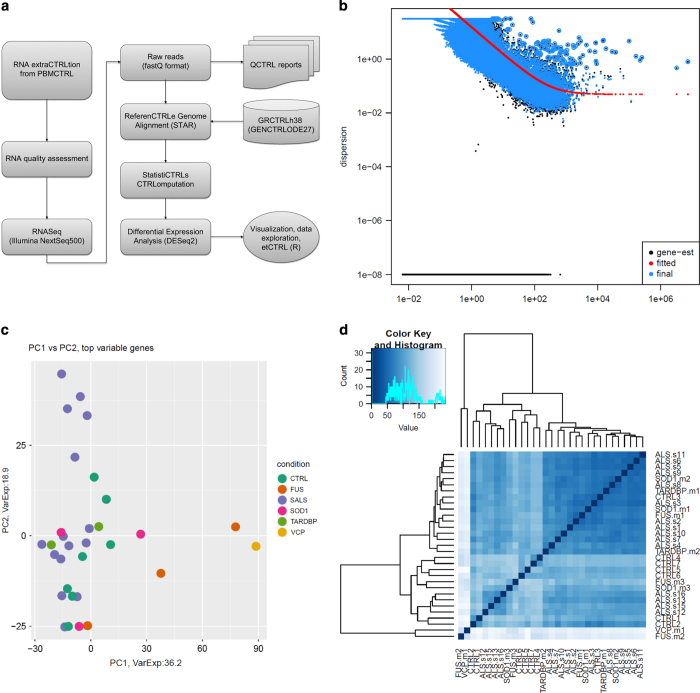
Experimental overiview and evaluation of sample variance. (**a**) The flowchart represents RNA-Seq workflow and data analysis. (**b**) An estimate of the dispersion parameter for each gene is shown. (**c**) Principal component analysis results. (**d**) Heatmap showing the sample-to-sample distance. It was obtained with DeSeq2 package on regularized-logarithm transformed counts. Color code is reported above the heatmap.

Quality of individual sequences were evaluated using FastQC software (see Code Availability 1) after adapter trimming with cutadapt software. Per base sequence quality plots, showing the Phred quality score distribution among all sample reads are shown in Figure 1. Gene and transcript intensities were computed using STAR/RSEM software^19^ using the “*--strandness forward*” option (see Code Availability 2 and 3 and Figure 2). Human genome reference used for the alignment was GRCh38 (Gencode release 27): in a rapidly evolving field like the one of non coding RNA analysis it is indeed fundamental to use up-to-date reference versions containing all the available information about annotated coding and non coding RNAs.

Mapping results are summarized in Table 2. Percentage of uniquely mapped reads is 21.8% on average, with standard deviation 10.35%. Remaining reads belong to ribosomal RNA because of the non perfect ribosomal RNA depletion. Since 10 to 25 M reads are suggested for RNA-Seq experiments^20^, this dataset is suitable for differential expression analysis, since about 60% of samples (21/36) have at least 10 M reads. Furthermore, biological replicates are the most effective strategy to improve statistical power in such experiments. For this reason, we decided to share our dataset with the scientific community, to have the possibility of integrating our data with similar data obtained in different laboratories.

**Figure 3:**
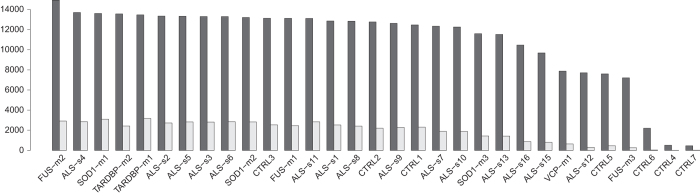
Detected transcripts per sample. Number of detected transcripts per sample for coding (dark grey bars) and lncRNAs (light grey bars), separately. Transcripts were considered if covered by at least 5 reads. Dark grey bars represent coding RNAs while light grey bars represent lncRNAs.

Expressed transcripts per sample were evaluated imposing a minimum threshold of 5 counts per gene to consider it as expressed. Figure 3 shows the amount of coding and non coding transcripts detected in each sample. With the exception of 4 samples which have a low number of detected transcripts (probably due to a lower number of available reads), 7000 to 14000 expressed coding genes and 500 to 3000 non coding genes were detected per sample. This is in accordance with the currently available knowledge about non coding transcripts that result to be globally less ex-pressed than coding ones within the cell^21^.

### Downstream analysis

Differential expression analysis for all transcripts (coding and non coding RNAs) was performed with the R package DESeq2^22^ (see Code Availability 4). This tool was selected because of its superior performance in identifying isoforms differential expression^23^.

Genes were considered differentially expressed and retained for further analysis with |log_2_(disease sample/healthy control)| ≥ 1 and a FDR ≤ 0.1. Analysis workflow is summarized in Figure 2.

For each sample, we evaluated the number of detected transcripts for coding and lncRNAs, separately. Transcripts were considered if covered by at least 5 reads^24^. Results are summarized in Figure 3.

### Code availability

The following software and versions were used for quality control and data analysis as described in the main text:

1. FastQC v0.11.6 was used for quality assessment of raw FASTQ sequencing data: http://www.bioinformatics.babraham.ac.uk/projects/fastqc/
2. STAR 2.6 was used for reads mapping to the human GRCh38 genome assembly: https://github.com/alexdobin/STAR/releases
3. RSEM v1.3.0, was used for gene expression quantification: http://bioinf.wehi.edu.au/featureCounts/
4. DESeq2 v1.20.0 was used for differential gene expression analysis: http://bioconductor.org/packages/DESeq2

## Data Records

Raw FASTQ files for the RNA-seq libraries were deposited to the NCBI Sequence Read Archive (SRA), and have been assigned BioProject accession PRJNA416880, Table 1, (Data Citation 1) and BioProject accession PRJNA474387, Table 1 (Data Citation 2). Raw counts data were deposited to the NCBI Gene Expression Omnibus (GEO) with accession number GSE106443, Table 1 (Data Citation 3) and GSE115259, Table 1 (Data Citation 4). First dataset has SRA SRP123453, second dataset has SRA SRP149638.

## Technical Validation

### RNA integrity assessment

RNAs were quantified using a Nanodrop ND-100 Spectrophotometer (Nanodrop Technologies, Wilmington, USA) and a 2100 Bioanalyzer (Agilent RNA 6000 Nano Kit, Waldbronn, Germany); RNAs with a 260:280 ratio of ≥1.5 and an RNA integrity number of ≥8 were subjected to deep sequencing.

### RNA-seq data qyality assessment

We applied FastQC v0.11.5 software to verify that per base quality scores are suitable for downstream analysis (Figure 1). Mapping percentage have been computed and are reported in Table 2. PCA plot, distance matrix and dispersion estimates are also shown in Figure 2.

## Additional information

**How to cite this article**: Zucca, S. *et al*. RNA-Seq profiling in Peripheral Blood Mononuclear Cells of Amyotrophic Lateral Sclerosis patients and controls. *Sci. Data*. 6:190006 https://doi.org/10.1038/sdata.2019.6 (2019).

**Publisher’s note**: Springer Nature remains neutral with regard to jurisdictional claims in published maps and institutional affiliations.

## Supplementary Material



Supplementary Figure 1

## Figures and Tables

**Table 1:** RNA-seq profiling to evaluate differential gene expression. All samples had the same source (blood sample) and were processed with the following steps: PBMCs isolation, RNA extraction and RNA-seq.

Sample	GEO	Sex	Age	Age of onset	Mutation
CTRL1	GSE106443	M	48	na	na
CTRL2	GSE106443	M	60	na	na
CTRL3	GSE106443	M	68	na	na
CTRL4	GSE115259	M	38	na	na
CTRL5	GSE115259	F	36	na	na
CTRL6	GSE115259	F	40	na	na
CTRL7	GSE115259	F	40	na	na
ALS-s1	GSE106443	M	66	65	na
ALS-s2	GSE106443	M	63	61	na
ALS-s3	GSE106443	F	61	60	na
ALS-s4	GSE106443	F	70	68	na
ALS-s5	GSE106443	M	56	55	na
ALS-s6	GSE106443	F	55	53	na
ALS-s7	GSE106443	F	56	55	na
ALS-s8	GSE106443	F	83	73	na
ALS-s9	GSE106443	M	66	60	na
ALS-s10	GSE106443	F	58	58	na
ALS-s11	GSE106443	M	68	65	na
ALS-s12	GSE115259	M	86	84	na
ALS-s13	GSE115259	F	68	65	na
ALS-s15	GSE115259	M	71	70	na
ALS-s16	GSE115259	F	69	67	na
FUS-m1	GSE106443	M	49	48	FUS R521C
FUS-m2	GSE106443	F	50	49	FUS P160R
FUS-m3	GSE115259	M	57	56	FUS G302A
SOD1-m1	GSE106443	M	50	49	SOD1 L106F
SOD1-m2	GSE106443	F	74	73	SOD1 G147S
SOD1-m3	GSE115259	F	58	57	SOD1 G93D
TARDBP-m1	GSE106443	F	52	50	TARDBP A382T
TARDBP-m2	GSE106443	M	68	66	TARDBP G357D
VCP-m1	GSE115259	M	51	50	VCP R191Q
All samples included in this study were Italian. All ALS patients had spinal onset and sample collection was performed after 6 months from exordium.					

**Table 2:** RNA-seq read statistics. All RNA samples used to perform RNA-Seq analaysis had RIN > = 8 and 260/280 ratio > 1.5, as recommended in manufacturer’s protocol.

Sample	Number of input reads	Average input read length	Uniquely mapped reads %	Number of reads mapped to multiple loci	% of reads mapped to multiple loci	Seq. batch
CTRL1	9.85E + 07	2 × 75	22.68%	5.55E + 07	56.38%	1
CTRL2	8.51E + 07	2 × 75	25.65%	5.49E + 07	64.53%	1
CTRL3	9.00E + 07	2 × 75	26.99%	5.53E + 07	61.47%	2
CTRL4	2.00E + 07	2 × 75	6.50%	1.23E + 07	61.53%	2
CTRL5	2.28E + 08	2 × 75	11.02%	1.71E + 08	74.93%	3
CTRL6	1.05E + 08	2 × 150	4.17%	2.88E + 07	27.54%	4
CTRL7	5.53E + 07	2 × 150	2.96%	1.77E + 07	31.95%	4
ALS-s1	8.30E + 07	2 × 75	26.69%	3.96E + 07	47.72%	1
ALS-s2	9.25E + 07	2 × 75	27.52%	5.73E + 07	61.92%	1
ALS-s3	9.39E + 07	2 × 75	33.88%	5.23E + 07	55.73%	1
ALS-s4	9.10E + 07	2 × 75	37.71%	2.56E + 07	28.17%	1
ALS-s5	9.18E + 07	2 × 75	31.32%	5.51E + 07	60.00%	2
ALS-s6	8.74E + 07	2 × 75	32.43%	5.29E + 07	60.55%	2
ALS-s7	1.03E + 08	2 × 75	17.58%	7.60E + 07	73.79%	2
ALS-s8	7.73E + 07	2 × 75	28.06%	4.71E + 07	60.97%	2
ALS-s9	7.32E + 07	2 × 75	24.14%	3.69E + 07	50.35%	3
ALS-s10	9.12E + 07	2 × 75	14.76%	5.79E + 07	63.49%	3
ALS-s11	7.83E + 07	2 × 75	34.72%	4.48E + 07	57.26%	3
ALS-s12	4.11E + 07	2 × 150	7.12%	2.12E + 07	51.64%	4
ALS-s13	7.17E + 07	2 × 150	17.87%	3.76E + 07	52.36%	4
ALS-s15	5.36E + 07	2 × 150	10.09%	3.52E + 07	65.63%	4
ALS-s16	8.36E + 07	2 × 150	7.68%	5.53E + 07	66.15%	4
FUS-m1	9.63E + 07	2 × 150	22.49%	6.84E + 07	71.03%	4
FUS-m2	9.82E + 07	2 × 75	13.62%	7.33E + 07	74.65%	2
FUS-m3	1.65E + 06	2 × 150	10.43%	1.28E + 07	81.24%	4
SOD1-m1	1.14E + 08	2 × 75	28.38%	6.86E + 07	60.15%	3
SOD1-m2	8.50E + 07	2 × 75	31.92%	4.88E + 07	57.46%	1
SOD1-m3	3.51E + 07	2 × 75	22.36%	2.33E + 07	66.42%	3
TARDBP-m1	1.16E + 08	2 × 75	31.21%	6.21E + 07	53.46%	3
TARDBP-m2	7.94E + 07	2 × 75	29.40%	3.50E + 07	44.14%	3
VCP-m1	2.98E + 07	2 × 75	13.46%	2.07E + 07	69.66%	1
