# Supplementary material for: RNA-Seq profiling in peripheral blood mononuclear cells of amyotrophic lateral sclerosis patients and controls
Source: Sci Data. 2019 Feb 5;6:190006. doi: 10.1038/sdata.2019.6 (PMC6362931; doi:10.1038/sdata.2019.6)
Supplement: Supplementary Figure 1 [file sdata20196-s2.pdf]

Supplementary Figure 1 : Bioanalyzer electropherograms of the libraries. Not available for CTRL5 and CTRL6.

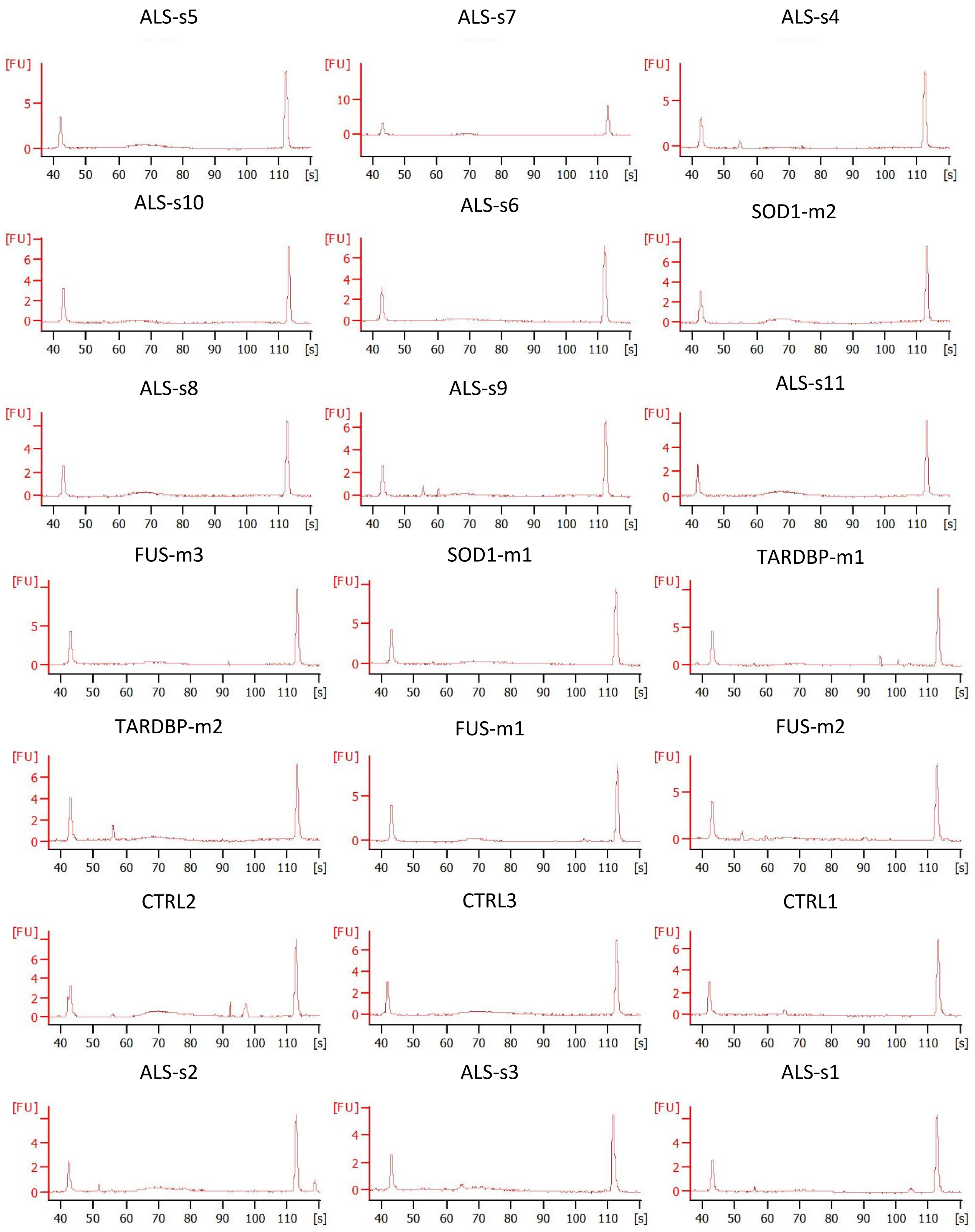

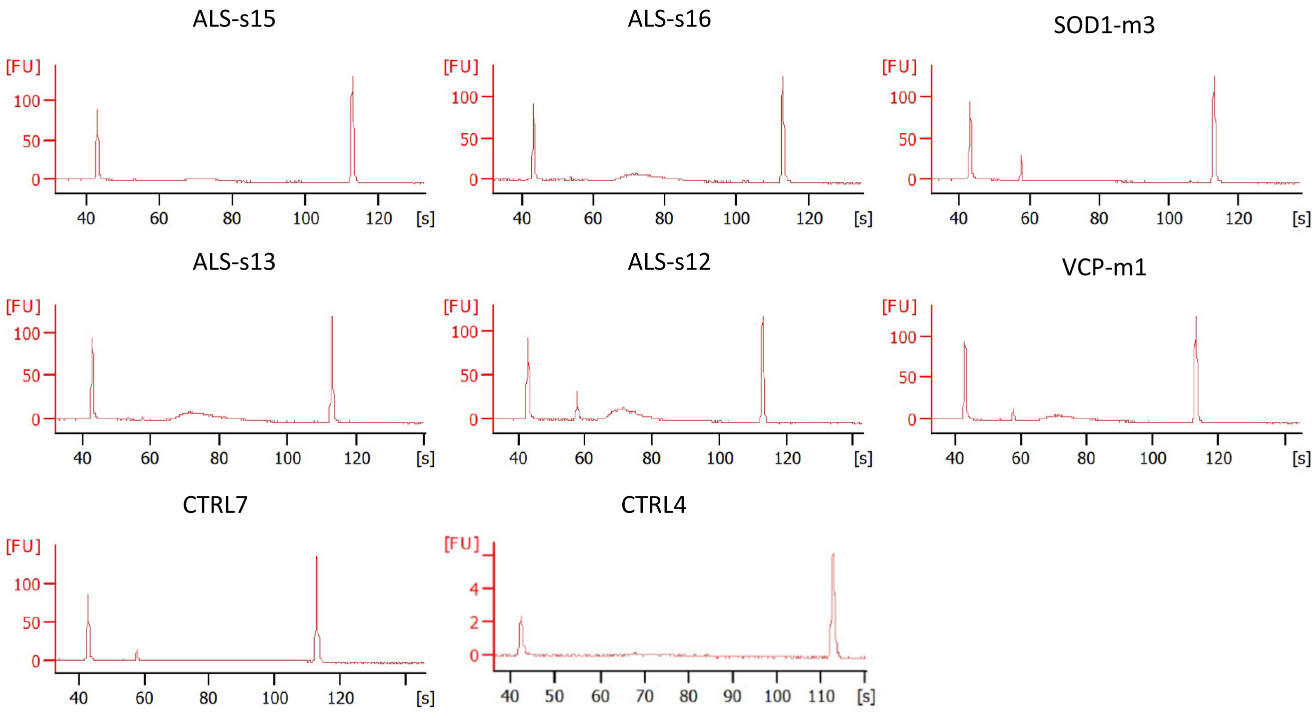

ALS-s15
[FU]
100
50
0
40 60 80 100 120 [s]
ALS-s16
[FU]
100
50
0
40 60 80 100 120 [s]
SOD1-m3
[FU]
100
50
0
40 60 80 100 120 [s]
ALS-s13
[FU]
100
50
0
40 60 80 100 120 [s]
ALS-s12
[FU]
100
50
0
40 60 80 100 120 [s]
VCP-m1
[FU]
100
50
0
40 60 80 100 120 [s]
CTRL7
[FU]
100
50
0
40 60 80 100 120 [s]
CTRL4
[FU]
4
2
0
40 50 60 70 80 90 100 110 [s]
